# Rotavirus Strain Distribution before and after Introducing Rotavirus Vaccine in India

**DOI:** 10.3390/pathogens10040416

**Published:** 2021-04-01

**Authors:** Tintu Varghese, Shainey Alokit Khakha, Sidhartha Giri, Nayana P. Nair, Manohar Badur, Geeta Gathwala, Sanjeev Chaudhury, Shayam Kaushik, Mrutunjay Dash, Nirmal K. Mohakud, Rajib K. Ray, Prasantajyoti Mohanty, Chethrapilly Purushothaman Girish Kumar, Seshadri Venkatasubramanian, Rashmi Arora, Venkata Raghava Mohan, Jacqueline E. Tate, Umesh D. Parashar, Gagandeep Kang

**Affiliations:** 1 The Wellcome Trust Research Laboratory, Division of Gastrointestinal Sciences, Christian Medical College, Vellore 632004, India; tintu.varghese@cmcvellore.ac.in (T.V.); shainey.rnc0411@gmail.com (S.A.K.); sidharthgiri@gmail.com (S.G.); nayana.arun@cmcvellore.ac.in (N.P.N.); 2 Department of Pediatrics, Sri Venkateshwara Medical College, Tirupati 517507, India; punya_manohar2002@yahoo.com; 3 Department of Pediatrics, Post Graduate Institute of Medical Sciences, Medical Road, Rohtak, Haryana 124001, India; geetagathwala@gmail.com; 4 Department of Pediatrics, Dr Rajendra Prasad Government Medical College, Tanda, Himachal Pradesh 176001, India; s_chaudhary@ymail.com; 5 Department of Pediatrics, Indira Gandhi Medical College, Shimla, Himachal Pradesh 171001, India; shayam.kaushik@live.in; 6 Department of Pediatrics, Institute of Medical Sciences and SUM Hospital, Bhubaneswar, Odisha 751003, India; m.dash74@gmail.com; 7 Department of Pediatrics, Kalinga Institute of Medical Sciences, 5 KIIT Road, Bhubaneswar, Odisha 751024, India; nkmohakud@yahoo.co.in; 8 Department of Pediatrics, Hi-Tech Hospital, Bhubaneswar, Odisha 751025, India; drrajib2007@gmail.com (R.K.R.); prasantij53@gmail.com (P.M.); 9 ICMR National Institute of Epidemiology, Chennai, Tamil Nadu 600077, India; girishmicro@gmail.com (C.P.G.K.); subramanianv89@yahoo.co.in (S.V.); 10 Translational Health Science and Technology Institute, Faridabad, Haryana 121001, India; arorarashmi2015@gmail.com; 11 Department of Community Health, Christian Medical College, Vellore 632002, India; venkat@cmcvellore.ac.in; 12 Centers for Disease Control and Prevention, Atlanta, GA 30333, USA; jqt8@cdc.gov (J.E.T.); uap2@cdc.gov (U.D.P.)

**Keywords:** rotavirus diarrhea, rotavirus genotyping, Rotavac vaccine

## Abstract

In April 2016, an indigenous monovalent rotavirus vaccine (Rotavac) was introduced to the National Immunization Program in India. Hospital-based surveillance for acute gastroenteritis was conducted in five sentinel sites from 2012 to 2020 to monitor the vaccine impact on various genotypes and the reduction in rotavirus positivity at each site. Stool samples collected from children under 5 years of age hospitalized with diarrhea were tested for group A rotavirus using a commercial enzyme immunoassay, and rotavirus strains were characterized by RT-PCR. The proportion of diarrhea hospitalizations attributable to rotavirus at the five sites declined from a range of 56–29.4% in pre-vaccine years to 34–12% in post-vaccine years. G1P[8] was the predominant strain in the pre-vaccination period, and G3P[8] was the most common in the post-vaccination period. Circulating patterns varied throughout the study period, and increased proportions of mixed genotypes were detected in the post-vaccination phase. Continuous long-term surveillance is essential to understand the diversity and immuno-epidemiological effects of rotavirus vaccination.

## 1. Introduction

Rotavirus is the leading etiology of acute gastroenteritis in children under 5 years old worldwide, causing high mortality, especially in middle- and low-income countries. India accounts for 22% of the total global rotavirus mortality [1]. In India, 40% of all diarrhea-related hospitalizations among children under 5 years of age is caused by group A rotavirus [2].

The genome of group A rotavirus is composed of 11 double-stranded RNA segments, of which the VP7 and VP4 genes coding for the outer capsid proteins are used for the classification of the virus into G and P types, respectively. Studies have been conducted across the globe to understand the natural evolution of rotavirus and its relevance in the context of vaccine introduction. Globally, G1P[8], G2P[4], G3P[8], and G9P[8] are the most common genotypes associated with rotavirus diarrhea [3]. However, it is hypothesized that large-scale vaccination may exert pressure on circulating strains, leading to possible changes in strain circulation.

In 2009, the World Health Organization (WHO) recommended the inclusion of rotavirus vaccines in the national immunization program of all countries. Currently, four live-attenuated oral vaccines are prequalified by WHO, which includes Rotarix (GlaxoSmithKline Biologicals, Rixensart, Belgium), RotaTeq (Merck & Co., Inc., West Point, PA, USA), Rotavac (Bharath Biotech, India), and Rotasiil (Serum Institute of India PVT. LTD., Pune, India). These rotavirus vaccines differ in their genotypic composition, with Rotarix and Rotavac being the monovalent vaccines and RotaTeq and Rotasiil being the pentavalent vaccines [4]. Rotarix and RotaTeq vaccines have been available on the market since 2006 and are currently used by nearly 90 countries in their immunization programs [4]. Early studies reported a decline in G1P[8] and the emergence of G2P[4] after Rotarix vaccination [5,6], while others showed no change [7]. The emergence of G9P[8] and G12P[8] was reported with the use of the RotaTeq vaccine [8]. However, such changes were also observed in other countries without rotavirus vaccination [9,10]. Hence, the vaccine impact on the circulating pattern of rotavirus strains is not clearly understood.

In India, the indigenously developed Rotavac vaccine, based on the human-bovine reassortant neonatal attenuated 116E strain, is a monovalent vaccine with the genotypic composition G9P [11]. It was introduced to the Universal Immunization Program (UIP) in April 2016 in a phased manner [11]. Other rotavirus vaccines like Rotateq and Rotarix were available in the private sectors for immunization before nationwide rotavirus vaccine implementation. India is the first Asian country to introduce rotavirus vaccines to the national immunization schedule, and currently, the Rotavac vaccine is used only in India and a few smaller countries [4]. The National Rotavirus Surveillance Network was established in India in 2005 to generate data on disease burden and monitor the trends of circulating genotypes [12,13]. This study describes the reduction in rotavirus prevalence and temporal trends in rotavirus strain distribution before and after Rotavac vaccine introduction in five sites in India.

## 2. Results

### 2.1. Prevalence of Rotavirus Diarrhea

Between September 2012 and June 2020, 8499 children under 5 years of age were enrolled in the surveillance study at the five sites. The details of enrollment and rotavirus testing are summarized in Table 1.

**Figure 1:**
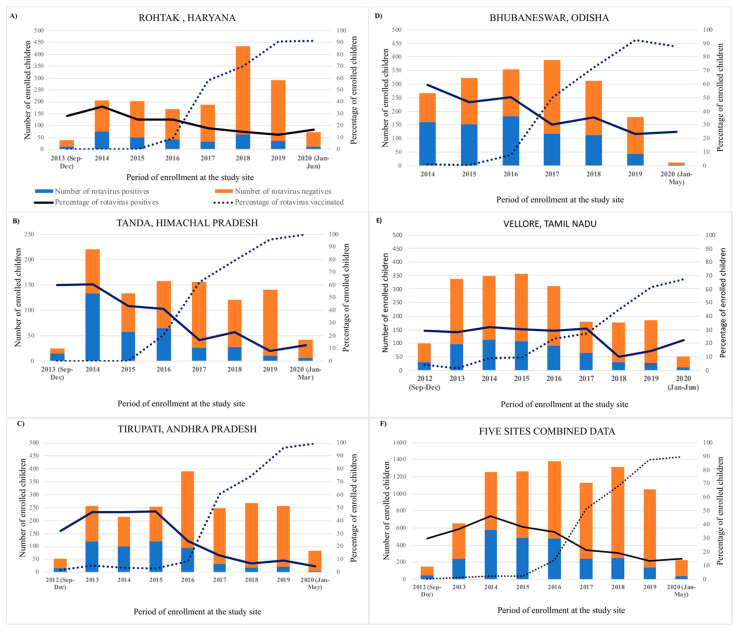
Impact of rotavirus vaccine after its introduction into the universal immunization programme in India, pre-vaccination and post-vaccination introduction surveillance comparison data from study sites at Rohtak (**A**), Tandak (**B**), Tirupati (**C**) Bhubaneswar (**D**), Vellore (**E**), and all the sites combined (**F**).

The proportion of diarrhea hospitalizations attributable to rotavirus at the five sites declined from a range of 56–29.4% in pre-vaccine years to 34–12% in post-vaccine years. The maximum annual positivity rate was in 2014 (46.2%), and the minimum was in 2019 (13.3%). The positivity rates declined steadily after vaccine implementation and were more marked towards the later years with higher vaccine coverage (Figure 1). The maximum reduction in rotavirus diarrhea was seen in Tirupati (72.1%), the site with maximum vaccine coverage, compared to a 32.5% reduction in Vellore, which was the last to introduce the vaccine and hence had the lowest overall vaccine coverage among the five sites.

### 2.2. Rotavirus Genotype Distribution in India

During the study period, genotyping was performed for 76.04% of the samples. The proportion of positive samples tested by genotyping PCR was greater in the post-vaccination period (97.02%) compared to the pre-vaccination period (64.97%), when the protocol changed for genotyping of a subset of samples.

**Figure 2:**
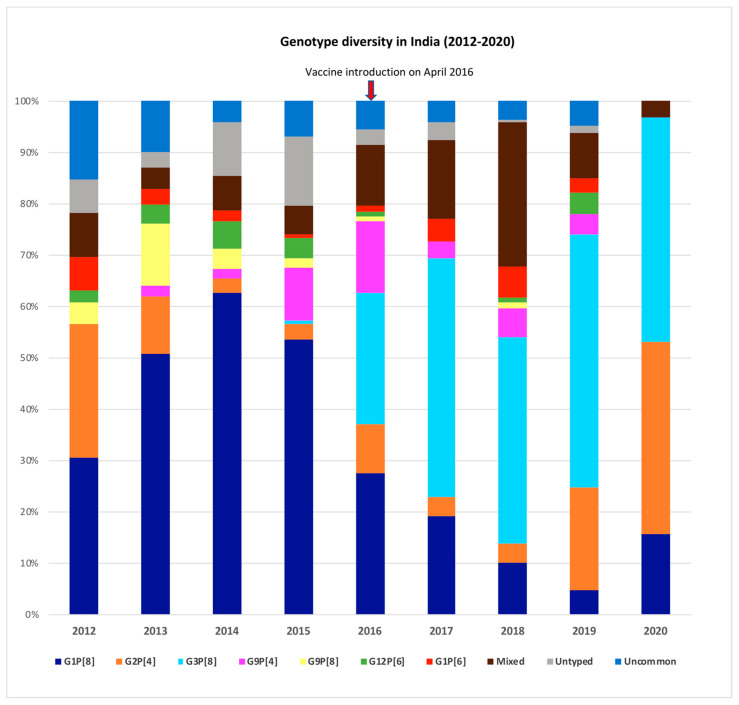
Data represented as the proportion of a specific genotype compared to the total genotype results. Uncommon genotype: <1% of total results; Mixed genotypes: those with >1 G or P-type; Untypables: those with either G or P untyped.

G1P[8] was the most common strain (49.5%) in the pre-vaccine period. The other common genotypes were G2P[4] (8%), G9P[4] (7.5%), G9P[8] (4.5%), and G12P[6] (3.8%). Conversely, G3P[8] (44.3%) was the most common genotype in the post-vaccine period, with G1P[8] (15.4%), G2P[4] (7.4%), G9P[4] (4.9%), and G1P[6] (3.7%) being the next most common genotypes (Figure 2). Marked yearly changes were seen among the circulating strains. Circulation of G9P[8] peaked during the year 2013, while G12P[6] increased in 2014/2015. Some reassortant strains like G1P[4], G2P[6], G2P[8], G3P[4], G3P[6], and G4P[6] were occasionally reported during the study period (Figure 2).

**Figure 3:**
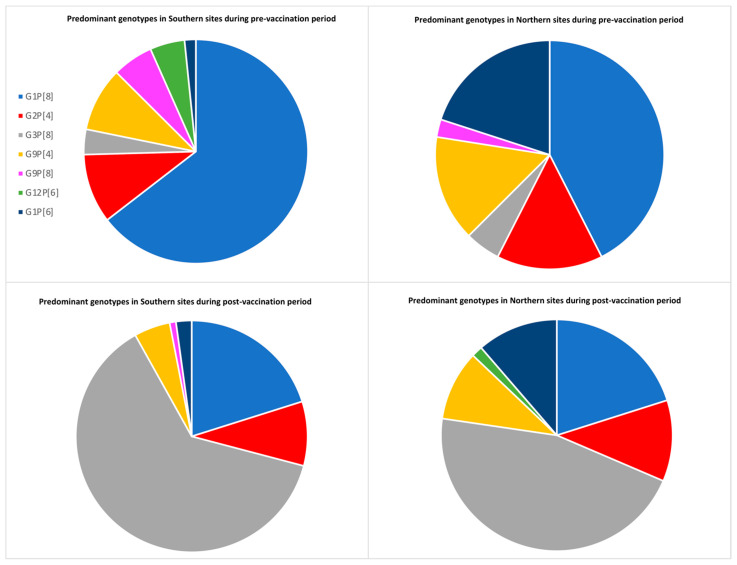
Comparison of genotype distribution between Northern sites (Tanda and Rohtak) and Southern sites (Vellore, Tirupati and Bhubaneswar). The major genotypes are compared during pre-vaccination (September 2012–April 2016) and post-vaccination period (May 2016–June 2020). The mixed genotype infections were excluded from the analysis.

The genotype distribution also varied across the sentinel sites in North India (Tanda and Rohtak) and South India (Vellore, Tirupati, and Bhubaneswar). G1P[6] was seen predominantly in northern sites, while G9P[8] and G12P[6] were seen in southern sites during the pre-vaccination period. In the post-vaccination period, the major circulating strains remained the same in northern sites, with G3P[8] topping the list. G3P[8] emerged in the southern sites as well, with a decline in G9P[8] and G12P[6] (Figure 3).

An increased prevalence of G3P[8] and decreased prevalence of G1P[8] were noted in the post-vaccination period compared to the pre-vaccination period. G1P[8] peaked during the year 2014 (62.6%) and has declined steadily since then. G3P[8] started appearing in 2015 and was the predominant genotype in the following years. No novel strains were detected during the post-vaccination period. Mixed genotype infections occurred in a higher proportion in the post-vaccine period (17.4%) compared to the pre-vaccine period (6.4%). G1 (33%) was the most common G-type found in mixed infections, mainly in combination with G12 (10.8%) and G3 (9%). Similarly, P[8] (93.4%) was the most common P-type in mixed infections, along with P[4] (55.6%) and P[6] (37.8%).

## 3. Discussion

Pre- and post-introduction surveillance at five sites in India indicate that vaccination is impacting severe rotavirus gastroenteritis. The overall prevalence of rotavirus in children with hospitalized gastroenteritis decreased after vaccine introduction, reaching 13.3% by the third year post-vaccine introduction, indicating the effectiveness predicted by clinical trials and modeling [14,15]. The maximum reduction rate was seen in Tirupati (72.1%), and the minimum was observed in Vellore (32.45%), which are the sites with maximum and minimum vaccine coverage, respectively.

During the study period, from 2012 to 2020, the major genotypes were G1P[8], G3P[8], G2P[4], G9P[4], G9P[8], G12P[6], and G1P[6], which include some reassortants that are not common in other parts of the world. There was marked temporal fluctuation, with G9P[8] detected at a high frequency in 2013/2014, only to disappear by 2019/2020, while G12P[6] was high in 2014/2015. Our findings are consistent with surveillance data from India and neighboring countries that also saw the emergence of G12 strains [16]. We also noted a geographic variation, with G12P[6] and G9P[8] seen more in the southern sites in 2012-2016 and G1P[6] observed more in the northern sites. These findings are in agreement with other studies conducted in the northern and southern parts of India [17,18,19,20]. The genotypic pattern in the northern sites had a rise in G3P[8] in the post-vaccine period compared to the pre-vaccine period, along with the disappearance of G9P[8] and the emergence of G12P[6]. However, the southern sites had a greater proportion of G3P[8] in the post-vaccination period, with a decline in both G12P[6] and G9P[8]. Variation in the geographic and temporal trends of rotavirus strains emphasizes the importance of multicentric studies.

Changes in genotype distribution and increased diversity are seen with other rotavirus vaccines. In Brazil, G2P[4] emerged as the major strain, while no change in genotype distribution was seen in Kenya after Rotarix introduction [7,21]. An increase in G3P[8] strain prevalence was seen in the United States after RotaTeq introduction [21,22]. In our study, G1P[8] was the predominant strain in the pre-vaccine period, coinciding with other studies conducted during this period [17,23,24], which declined thereafter with the emergence of G3P[8]. However, the rise in G3P[8] in 2017/2018 is likely to be a natural fluctuation rather than the effect of the vaccine, as there was a similar trend seen in other countries without rotavirus immunization [25,26]. There are currently ongoing efforts to examine rotavirus vaccine effectiveness against diseases caused by specific strains, which will help further address this issue.

Globally, the rate of mixed rotavirus infections is similar to our findings [23,27]. Mixed rotavirus infections can facilitate the evolution of novel strains by genetic reassortment between the segmented genes of rotavirus, eventually increasing its diversity. Other studies have reported an increased frequency of unusual and novel strains in the post-vaccine surveillance period [24,28]. In our study, reassortant strains including G1P[4], G2P[6], G3P[6], G3P[4], and G4P[6] were occasionally seen, with no specific increase in the post-vaccination phase. Whole-genome sequencing and phylogenetic analysis will help to identify possible reassortment of rotavirus genes and detect mutational events. This will help us in tracking the virus evolution over time, which might give us more insight into the drivers of viral strain circulation and the impact of vaccines.

To conclude, our study showed a reduction in rotavirus diarrhea across five sites in India after Rotavac vaccine introduction. Changes in circulating strains with an increased rate of mixed infections were also seen in the post-vaccine period. In our study from 2016, additional methods were used for the genotyping of samples that remained untyped with standard laboratory protocols. Some of the differences in genotypes before and after vaccination introduction may have been caused by the change in genotyping methods. Due to the short period of surveillance, it is difficult to determine whether the changes were due to natural strain variations or vaccine pressure. Continued surveillance is warranted to determine the long-term effects of rotavirus vaccination.

## 4. Materials and Methods

### 4.1. Study Sites

Active hospital-based surveillance for diarrhea was established in five sentinel sites consisting of major referral hospitals from September 2012 to June 2020. The hospitals included were Christian Medical College (Vellore, Tamil Nadu), Sri Venkateshwara Medical College (Tirupati, Andhra Pradesh), Hi-Tech hospital (Bhubaneswar, Odisha), Pt. Bhagwat Dayal Sharma Postgraduate Institute of Medical Sciences (Rohtak, Haryana), and Rajendra Prasad Government Medical College (Tanda, Himachal Pradesh).

### 4.2. Sample Collection and Laboratory Testing

Sample collection and laboratory methods are detailed in the study protocol [2]. In brief, children under 5 years of age hospitalized with diarrhea were enrolled in the study. A stool sample, vaccination card copy, and case report form with clinical and demographic details were collected from each child. Samples were stored at the appropriate temperature until transported to CMC, Vellore, which served as the main testing laboratory. All testing was done as per the modified WHO generic protocol for rotavirus surveillance [29]. Stool samples were screened for rotavirus VP6 antigen using a commercial enzyme immunoassay (EIA). All EIA positive samples were further characterized by reverse transcription-polymerase chain reaction (RT-PCR) for VP7 (G Type) and VP4 (P Type) genes. In brief, RNA was extracted from 20% fecal suspension using the QIAamp Viral RNA Mini Kit (Qiagen). Complementary DNA (cDNA) synthesized by reverse transcription using Moloney murine reverse transcriptase enzyme (Superscript II MMLV-RT, Invitrogen) and random primers (Invitrogen) were used as templates for VP7 and VP4 typing by a hemi-nested multiplex PCR using published primers [30,31]. For the samples collected in the post-vaccination period, additional typing methods were used if they remained untyped with standard laboratory testing protocols [32]. The negative samples by genotyping PCR were confirmed for rotavirus positivity by VP6 PCR [32]. The untyped samples and unusual rotavirus strains were sequenced by the Sanger sequencing method.

## Figures and Tables

**Table 1:** Enrollment and rotavirus testing details from 5 surveillance sites (September 2012–June 2020).

Site Name	Pre- Vaccination Period and Enrollment	Pre- Vaccination Rotavirus Positivity	Post- Vaccination Period and Enrollment	Post- Vaccination Rotavirus Positivity	Percentage Reduction in Rotavirus Positivity
Rohtak	489	153 (31.2%)	1103	169 (15.3%)	50.96%
Tanda	423	237 (56.0%)	573	104 (18.1%)	67.67%
Tirupati	930	401 (43.1%)	1089	131 (12.0%)	72.15%
Bhubaneswar	723	395 (54.6%)	1113	379 (34.0%)	37.72%
Vellore	1598	470 (29.4%)	458	91 (19.8%)	32.65%
Total	4163	1656 (39.7%)	4336	874 (20.1%)	49.37%

## Data Availability

Since the study is continuing in some settings, the data are still being generated and have not yet been placed in a public repository. The data analyzed during the period reported will be made available on request after de-identification.
